# Why we do what we do: a survey of ID providers about oral antibiotics

**DOI:** 10.1017/ash.2026.10421

**Published:** 2026-06-01

**Authors:** Mildred Nelson, Marisa Winkler, Sujit Suchindran, Lucy Sabrin Witt

**Affiliations:** 1 Emory University School of Medicine, USA; 2 JMI Laboratories, USA

## Abstract

We surveyed infectious disease providers to examine beliefs and practices around oral antibiotic use. Provider demographics did not predict beliefs or practices. Certain clinical scenarios were more likely to achieve consensus for oral prescribing. Understanding beliefs and practice patterns can help stewardship teams craft interventions to increase oral antibiotic use.

## Introduction

Evidence supports oral (PO) antibiotics as efficacious for definitive therapy in a variety of infectious diseases (IDs).^1,2^ Even morbid and challenging infectious syndromes (eg, *Staphylococcus aureus* bacteremia, endocarditis, prosthetic joint infections [PJI]) have been shown to be successfully treated with oral antibiotics.^3–6^ The risks of prolonged intravenous (IV) catheterization, including additional infections, blood clots, and increased length of stay, alongside scientific data supporting the efficacy of oral antibiotics make oral antibiotics the superior choice in many clinical scenarios.^1^ Despite this, ID clinicians hesitate to utilize oral antimicrobials for certain infections. These practice variations provide opportunities for stewardship teams to optimize oral antibiotic use and improve patient care.

To understand current practices among ID providers in our healthcare system, we conducted a survey to describe comfort level and beliefs around PO antibiotics as well as to test provider practice patterns. We sought to identify demographic variables that might predict the use of PO antibiotics over IV, understand what clinical scenarios PO antibiotics are more likely to be selected, and understand ID providers’ feelings around oral antibiotic use to enhance our stewardship efforts.

## Methods

We distributed an anonymous, Qualtrics™ survey (Supplement) via email to 137 Emory ID affiliated providers in February and March 2024 at five metropolitan Atlanta area hospitals within one healthcare system. The survey first collected respondents’ demographic information, practice characteristics (academic, including an affiliated Veterans Affairs hospital, vs private), years-since-training, medical role (attending, fellow, advanced practice provider, role or training in stewardship), followed by beliefs regarding oral antibiotics. The second section of the survey consisted of five clinical scenarios for which respondents were asked to choose definitive therapy from a selection of IV and PO options. To minimize bias, ID providers were not given details of the survey’s goals prior to response. Respondents were categorized as either high-or low-oral antibiotic prescribers (HOAP or LOAP) based upon how frequently they chose to use oral antibiotics in the five clinical scenarios provided; HOAP providers chose oral antibiotics in at least three of the five scenarios and LOAP providers chose to use antibiotics in fewer than three scenarios.

We evaluated all demographic variables, self-reported comfort levels and estimations of PO antibiotic use for association with HOAP or LOAP patterns using student’s *t*-test and χ^2^ or Fischer’s exact test when appropriate. Finally, we compared provider self-reported reasons to not choose PO antibiotics between HOAP and LOAP. SAS 9.4 Cary, NC was used for all analyses. This study was approved by the Emory University IRB.

## Results

A total of 60 clinicians out of 137 emailed (44%) completed the survey. A majority were attending physicians (Table 1). The largest age demographic was 31–40 years old (62%), 51.7% of respondents were either current trainees or were fewer than five years out of training while 35% of respondents were >11 years out of training. 87% of respondents self-identified as academic affiliated (52/60) and 13% (8/60) as private practice. Only 11% held a role or had received formal training in stewardship. Half of respondents were categorized as LOAP; 29 respondents chose PO antibiotics in two of the five scenarios, and one chose oral antibiotics in only one scenario (Supplement Table 1).

**Table 1.** Demographics of surveyed infectious disease clinicians as well as beliefs and practices around oral antibiotics, by high and low prescribers Table 1 long description.

	Total (n = 60)	Low oral prescribers (n = 30)	High oral prescribers (n = 30)	*P*-value
	N (%)	N (%)	N (%)	
**Age**				.32
20–30 yo	1 (1.7)	1 (3.3)	0 (0)	
31–40 yo	30 (50.9)	12 (40.0)	18 (62.1)	
41–50 yo	16 (27.1)	9 (30)	7 (24.1)	
51–60 yo	10 (17.0)	6 (20)	4 (13.8)	
>61 yo	2 (3.4)	2 (6.7)	0 (0)	
**Female**	30 (50)	14 (46.7)	16 (53.3)	.84
**Type of practice (academic v other)**				<.01
Academic	52 (86.7)	22 (73.3)	30 (100)	
Veterans healthcare associated	4 (6.7)	2 (6.7)	2 (6.7)	
Non-academic	8 (13.3)	8 (26.7)	0 (0)	
**Years since training**				.46
Current trainee	13 (21.7)	4 (13.3)	9 (30.0)	
0–5 years	18 (30.0)	10 (33.3)	8 (26.7)	
6–10 years	8 (13.3)	4 (13.3)	4 (13.3)	
≥11 years	21 (35.0)	12 (40.0)	9 (30.0)	
**Current role**				.21
Fellow	13 (21.7)	4 (13.3)	9 (30.0)	
Attending physician	43 (71.7)	23 (76.7)	20 (66.7)	
APP	4 (6.7)	3 (10.0)	1 (3.3)	
**Holds a formal role or received formal training in antimicrobial stewardship**	7 (11.7)	5 (16.7)	2 (6.7)	.42
**Which of the following is a major reason you do NOT use oral antibiotics** as step-down therapy for serious infections*				
Concern that oral antibiotics will not effectively penetrate the infected tissue	41 (68.3)	22 (73.3)	19 (63.3)	
Concern that oral antibiotics are not as “powerful”	3 (5.0)	1 (3.3)	2 (6.7)	
Concern that patients will perceive oral antibiotics as not being as effective	6 (10.0)	3 (10.0)	3 (10.0)	
Concern that using oral antibiotics is not evidence based	15 (25.0)	10 (33.3)	5 (16.7)	
Concern about litigation if re-infection occurs	9 (15.0)	6 (20.0)	3 (10.0)	
Concern that my colleagues will not approve of oral antibiotic use	7 (11.7)	4 (13.3)	3 (10.0)	
Concern that patients on oral antibiotics do not follow up in clinic compared to OPAT patients	16 (26.7)	9 (30.0)	7 (23.3)	
My supervisor(s) do not want me to use oral antibiotics	9 (15.0)	1 (3.3)	8 (26.7)	
I have financial incentives to use IV antibiotics	0 (0)	0 (0)	0 (0)	
I have no concerns about using oral antibiotics	9 (15.3)	5 (16.7)	4 (13.3)	

Note. APP, advanced practice provider; OPAT, outpatient parenteral antimicrobial therapy.

*Could choose multiple answers.

Age—1 missing, Gender—3 preferred not to say.

### Feelings and beliefs about PO antibiotics

**Figure 1.**
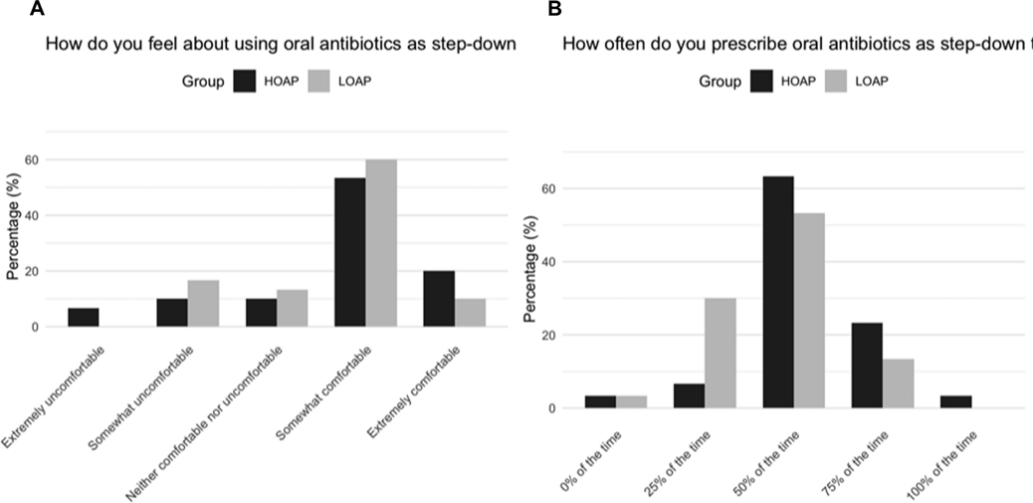
(a) How high and low prescribers feel about oral antimicrobial therapy (b) How providers believe they use oral antibiotic therapy as step-down. HOAP, high oral antibiotic prescribers; LOAP, low oral antibiotic prescribers. Figure 1 long description.

The most reported concern regarding using oral antibiotics involved penetration into affected tissue, for a majority of both HOAP (63.3%) and LOAP (73.3%). HOAP were more likely to cite supervisor concerns with oral antibiotics as a barrier to prescribing. Similar rates of HOAP and LOAP expressed feeling “Somewhat” or “Extremely” comfortable prescribing PO antibiotics for definitive therapy (Figure 1). Most providers estimated they prescribed PO antibiotics around 50% of the time, with HOAP providers more likely to estimate they prescribe PO antibiotics ≥75% of the time (Figure 1).

### Clinical cases

All providers chose oral step-down therapy for *E. coli* bacteremia (Supplement Table 1). Over 98% of providers chose oral antibiotics in a patient with a diabetic foot ulcer whose cultures grew MRSA and had achieved source control (60/60 HOAP and 59/60 LOAP). Conversely, only a single provider chose an oral option for step-down therapy for left-sided native valve endocarditis caused by *E. faecalis.* For line-associated methicillin-susceptible *Staphylococcus aureus* (MSSA) bacteremia (with line removed) 73.3% (22/30) of HOAP chose oral antibiotics as definitive therapy compared to no LOAP. Similarly, 43.3% (13/30) of HOAP chose oral antibiotics as destination therapy for a patient with MSSA PJI post debridement and retention as compared to no LOAP.

### Predicting HOAP or LOAP

Age, clinical role, years since training, and stewardship training or responsibilities were not associated with frequency of oral antibiotic prescribing in clinical scenarios (Table 1). The only statistically significant association with LOAP was the type of practice with all private practice classified as LOAP.

## Discussion

We aimed to identify demographic, behavioral, and clinical determinants of antimicrobial prescribing practices that could be targeted by our antimicrobial stewardship program (ASPs). Interestingly, age, years from training, and current role did not predict HOAP/LOAP categorization, contrasting prior work suggesting senior physicians may rely on past experience to guide treatment regimens.^7^ The reason for private practice providers’ preference for IV antibiotic is likely multifactorial; historic practice patterns, cultural norms, and potential motivation for IV antibiotic prescribing as some may rely on revenue from privately owned infusion centers.^8^ Concern about litigation and colleagues’ approval was low for all providers.

There was consensus across HOAP and LOAP in some clinical scenarios. In uncomplicated Gram-negative bacteremia, all providers favored PO antibiotics. Our ASP created and implemented guidelines on oral transitions for uncomplicated Gram-negative bacteremia in 2024, bolstered by the data supporting this practice and clear agreement amongst all ID providers in our system. Conversely, MSSA catheter-related bacteremia and a PJI were managed differently even within HOAP suggesting that opportunities exist for management of these scenarios with oral antibiotics. An earlier survey of ID providers showed similarly less comfort with oral antibiotics for *Staphylococcus aureus* bacteremia compared to Gram-negative infections.^9^ Interestingly, almost no providers were comfortable with oral antibiotics for *Enterococcus endocarditis* despite evidence of non-inferiority reported by the POET trial.^3^ This suggests that more data may be required before clinicians can be convinced to use oral antibiotics in endocarditis and may discourage ASP efforts focused on promoting such a change.

HOAP providers self-estimated higher PO antibiotic use than LOAP providers suggesting appropriate self-awareness of preference for PO versus IV. Previous survey studies have shown discordance between practice patterns and self-estimated habits.^10^ Providers could be categorized according to preference for oral prescribing and targeted with specific ASP interventions or education based on self-described habits.

Our data has limitations. The survey was only sent to providers within a regional healthcare system, and only a limited number of private practice physicians responded, limiting generalizability. Furthermore, clinicians’ responses to the provided clinical scenarios may not reflect true prescribing habits. Lastly, ID providers may not be directly involved in some of the clinical scenarios described, and as a result, their preferences may not provide meaningful insight to guide ASPs efforts in these scenarios.

Opportunities exist for targeted stewardship efforts to increase the use of oral antibiotics recommended by ID providers. Specific scenarios or dogma can be addressed to impact practice patterns and improve patient care using the existing evidence base.

## Supporting information

10.1017/ash.2026.10421.sm001 Nelson et al. supplementary material Nelson et al. supplementary material

## Data Availability

This data can be made available upon request and if approved by Emory University.

## Supplementary material

The supplementary material for this article can be found at https://doi.org/10.1017/ash.2026.10421.

## Table 1. Long description

The table presents data on the demographics and practices of infectious disease clinicians, categorized by high and low prescribers of oral antibiotics. It includes information on the age distribution, training status, practice type, and stewardship roles of the clinicians. The table has four rows and three columns, with headers for demographics, beliefs, and practices. Notable trends include a majority of clinicians being in the 31-40 age range, many being in academic settings, and a significant portion being recent trainees. The table also highlights the proportion of clinicians categorized as low or high prescribers of oral antibiotics.

Navigate back to Table 1..

## Figure 1. Long description

The bar graph compares two groups, high oral antibiotic prescribers (HOAP) and low oral antibiotic prescribers (LOAP), across two dimensions. The first graph (A) shows how comfortable each group feels about using oral antibiotics as step-down therapy. The x-axis lists comfort levels: extremely uncomfortable, somewhat uncomfortable, neither comfortable nor uncomfortable, somewhat comfortable, and extremely comfortable. The y-axis represents the percentage of respondents. HOAP and LOAP groups show varying comfort levels, with both groups having the highest percentage in the somewhat comfortable category. The second graph (B) illustrates how often each group prescribes oral antibiotics as step-down therapy. The x-axis indicates frequency categories: 0 percent of the time, 25 percent of the time, 50 percent of the time, 75 percent of the time, and 100 percent of the time. The y-axis shows the percentage of respondents. HOAP has the highest percentage in the 50 percent of the time category, while LOAP has a more distributed spread across the categories. All values are approximated.

Navigate back to Figure 1..
