# Supplementary material for: Why we do what we do: a survey of ID providers about oral antibiotics
Source: Antimicrob Steward Healthc Epidemiol. 2026 Jun 1;6(1):e159. doi: 10.1017/ash.2026.10421 (PMC13227119; doi:10.1017/ash.2026.10421)
Supplement: Nelson et al. supplementary material [file S2732494X26104215sup001.pdf]

Supplement

Supplement Table 1. Antibiotic Selection for Clinical Scenarios by High or Low Prescribers

	Total (n = 60)	**Low Oral Prescribers (n = 30)**	**High Oral Prescribers (n = 30)**
	N (%)	**N (%)**	**N (%)**
What is your ideal choice for destination/discharge antibiotic therapy for ***E. coli* bacteremia**, pan-susceptible, assuming listed as susceptible to all choices?			
IV Ceftriaxone	0 (0)		
IV Ertapenem	0 (0)		
Oral beta-lactam	11 (18.3)		
Oral TMP-SMX	8 (13.3)		
Oral Fluoroquinolone	41 (68.3)		
Prescribed Oral (Total)	60 (100)	30 (100)	30 (100)
What is your ideal choice for destination/discharge antibiotic therapy for uncomplicated **MSSA bacteremia – CLABS**I, one set positive, cleared within 24 hours, line source removed assuming listed as susceptible to all choices?			
IV Cefazolin	33 (55.0)		
IV Vancomycin	1 (1.7)		
IV Daptomycin	0 (0)		
IV Dalbavancin	4 (6.7)		
Oral Linezolid	22 (36.7)		
Prescribed Oral (Total)	22 (36.7)	0 (0)	22 (73.3)
What is your ideal choice for destination/discharge antibiotic therapy for **diabetic foot infection** (no bacteremia), source controlled, operative cultures grew **MRSA** assuming listed as susceptible to all choices?			
IV Vancomycin	0 (0)		
IV Daptomycin	0 (0)		
IV Dalbavancin	1 (1.7)		
Oral Linezolid	15 (25.0)		
Oral TMP-SMX	22 (37.7)		
Oral Doxycycline	22 (37.7)		
Prescribed Oral (Total)	59 (98.3)	29 (96.7)	30 (100)

What is your ideal choice for destination/discharge antibiotic therapy for uncomplicated **Enterococcus faecalis left-sided, native-valve, endocarditis** with bacteremia (cleared) assuming listed as susceptible to all choices? *			
IV Ampicillin + Gentamicin	4 (6.8)		
IV Ampicillin + Ceftriaxone	54 (91.5)		
Oral Amoxicillin + Rifampin	1 (1.7)		
Oral Linezolid + Rifampin	0 (0)		
Prescribed Oral (Total)	1 (1.7)	0 (0)	1 (3.3)
What is your ideal choice for destination/discharge antibiotic therapy for **prosthetic joint infection status-post one stage debridement and implant retention, culture growing MSSA** assuming listed as susceptible to all choices?			
IV Cefazolin + Rifampin	43 (71.7)		
IV Nafcillin + Rifampin	4 (6.7)		
Oral Levofloxacin + Rifampin	7 (11.7)		
Oral Doxycycline + Rifampin	1 (1.7)		
Oral Cefadroxil + Rifampin	5 (8.3)		
Prescribed Oral (Total)	13 (21.7)	0 (0)	13 (43.3)

E faecalis = 1 missing

Abbreviation: MSSA – methicillin susceptible Staphylococcus aureus, MRSA – methicillin resistant Staphylococcus aureus

Antibiotic Survey

Screening Questions

Inclusion/Exclusion

1. **Are you an infectious disease clinician who works at an Emory-associated facility?**

Y/N --> If no stop survey

2. **Do you currently care for patients as part of your work?**

Y/N --> If no stop survey

Demographics

3. **Which best describes your role?**

Physician / Advanced Practice Provider (Nurse Practitioner or Physician Assistant)/ Fellow/ Other

4. **Which best describes your type of practice?**

Private practice /Academic / Veterans Administration Associated / Kaiser Permanente/Currently in training

5. **Where do you see patients…**

Inpatient only/Outpatient only/Both inpatient and outpatient

6. **At which of these sites do you spend your in-patient clinical time (check all that apply)?**

Emory University Hospital/ Emory University Hospital Midtown/Emory Johns Creek Hospital/ Emory St Josephs Hospital/Emory Decatur Hospital/Emory University Orthopedic and Spine Hospital/Veterans Administration Medical Center/ Grady/ I do not practice inpatient

Clinical Scenario Questions

What is your ideal choice for <u>destination/discharge</u> antibiotic therapy, assuming listed as susceptible to all choices? Assume normal renal function, clinical improvement, and no contraindications to any of the listed regimens.

E coli bacteremia, pan-susceptible
- Ceftriaxone
- Ertapenem
- Oral beta lactam
- Oral TMP-SMX
- Oral fluoroquinolones

Uncomplicated MSSA bacteremia – CLABSI, one set positive, cleared within 24 hours, line source removed
- IV Cefazolin
- IV Vancomycin

- IV Daptomycin
- IV Dalbavancin
- Oral linezolid

Diabetic foot infection (no bacteremia), source controlled, operative cultures grew MRSA.
- IV Vancomycin
- IV Daptomycin
- IV Dalbavancin
- Oral linezolid
- Oral TMP-SMX
- Oral doxycycline

Enterococcus faecalis left-sided, native-valve, endocarditis with bacteremia (cleared)
- IV Ampicillin + gentamicin
- IV Ampicillin + ceftriaxone
- Oral Amoxicillin + rifampin
- Oral Linezolid + rifampin

Prosthetic joint infection status-post one stage debridement and implant retention, culture growing MSSA
- IV cefazolin + rifampin
- IV nafcillin + rifampin
- oral levofloxacin + rifampin
- oral doxycycline + rifampin
- oral cefadroxil + rifampin

Qualitative Questions

On a scale of 1-5 (5 being very comfortable, 1 being very uncomfortable), how do you feel about using oral antibiotics as step-down therapy for serious infections?
1 2 3 4 5

How often do you prescribe oral antibiotics as step-down therapy for serious infections?
0% of the time
25% of the time
50% of the time
75% of the time
100% of the time

Which of the following is a major reason you do NOT use oral antibiotics as step-down therapy for serious infections (choose all that apply)?
Concern that oral antibiotics will not effectively penetrate into the infected tissue
Concern that oral antibiotics are not as “powerful” as IV antibiotics
Concern that patients will perceive oral antibiotics as not being as effective as IV antibiotics

Concern that using oral antibiotics is not evidence based
Concern about litigation if re-infection occurs
Concern that my colleagues will not approve of oral antibiotic use
Concern that patients on oral antibiotics do not follow up in clinic compared to OPAT patients
My supervisor(s) do not want me to use oral antibiotics
I have financial incentives to use IV antibiotics
I have no concerns about using oral antibiotics

Demographics Part 2

1. **How many years have you been practicing Infectious Diseases (beginning after fellowship)?**

Current Fellow / 0-5 years / 6-10 years / >11 years

2. **Do you have a formal stewardship role or formal training in Antimicrobial Stewardship (such as a SHEA course)?**

Y/N
